# Care Navigation and Coordination Program on Reducing Hospital Use for Adults with Complex Health and Psychosocial Needs in South West Sydney, Australia

**DOI:** 10.5334/ijic.7739

**Published:** 2024-07-10

**Authors:** Anita Hartati, Madison Jarrett, Brendon McDougall, Megan Kent, Maja Ljubojevic, Kylie Stolzenhein

**Affiliations:** 1 Keeping Well in Community, Primary and Community Health, South Western Sydney Local Health District, AU; 2 Keeping Well in Community, Primary and Community Health, South Western Sydney Local Health District, US; 3 Primary and Community Health, South Western Sydney Local Health District, AU

**Keywords:** integrated care, care navigation and coordination, chronic disease, hospital utilization, complex needs, program evaluation

## Abstract

**Intro:**

Complex and siloed health and social service systems can be difficult for people to navigate. The fragmented and poorly linked services leads to ineffective communication between care teams, delayed access to services, concerns regarding quality and safety of patient care, as well as patient frustration and disengagement.

**Description:**

Planned Care for Better Health (PCBH) is a community-based care navigation and coordination program for people with complex health and psychosocial needs who are at risk of future hospitalisation. It focuses on early identification and holistic care to remove barriers and improve access to healthcare. By including a persons’, family and carers in planning, listening to their needs, supporting the person to achieve their goals, and empowering them to make decisions on their own health, PCBH aims to enhance clients’ healthcare experience and reduce preventable hospital utilisation.

**Discussion:**

Building trusting and collaborative relationships with clients, families, carers, and health service providers requires commitment. Acknowledging and addressing psychosocial needs is critical for enhancing health outcomes. Equipping patients with self-management skills and knowledge to navigate and engage support services may generate lasting effects, even post-program enrolment.

**Conclusion:**

PCBH is associated with a notable reduction in unplanned hospitalisations and total bed days. However, reduction in ED presentations is similar between the intervention and comparison cohorts. Future initiatives should focus on a shared vision of integrated care, robust leadership, and participative co-creation with service-level stakeholders. Sustained program establishment, a multidisciplinary care coordinator team, and an early creation of robust evaluation strategy must be considered.

## Introduction

Care integration across various sectors and providers has become a goal in the healthcare landscape as a strategic response to the increasing burden of chronic diseases and the substantial impact of related psychosocial needs on their management. Chronic conditions are the leading causes of illness, disability and death in Australia. People often face compounded complexity such as reduced capacity to participate, competing priorities, and inequitable access [1], which stem from psychosocial situations such as mental illness, disability, limited literacy, or cultural and linguistic diversity, thus increasing susceptibility to poor health outcomes [2,3,4].

The Australian healthcare and social services system is known for its complexity [1,5], siloed by geographical areas, specialties, professional networks, or levels of government [6,7], hence complicating patient navigation. This results in uncoordinated care and delayed services, ineffective communication between care teams, and concern regarding the quality and safety of patient care. This leads to patient frustration, disempowerment, and disengagement [8]. The healthcare system is thus falling short for people with chronic conditions who are twice as likely to require hospital admission and endure disproportionately longer hospital stays [9], with a higher likelihood of readmission following discharge [10]. People with chronic conditions contribute significantly to potentially preventable hospitalisations (PPH), a metric often used as an indirect measure of primary healthcare effectiveness, but also a powerful tool to investigate health inequalities and identify variation among population groups [11]. In 2017–2018, chronic conditions accounted for 46% of all PPH in Australia [11], costing the healthcare system over $320 million annually [12]. Hospitalisation for this population is a multifaceted problem that not only reflects the severity of the diseases but also the complexity of their unmet psychosocial needs, healthcare system attributes, and wider determinants of health [10,13]. International organisations have advocated for integrated services across the whole spectrum of health and social care to improve health outcomes while at the same time delivering a higher quality service to patients, lowering costs and ensuring the wellbeing of the health workforce [5,14]. Yet, in Australia, changes in practice have not kept up with policy intentions nor has practice adequately engaged patients in ways that are inclusive of their psychosocial needs [15,16].

Systematic reviews have shown that care navigation and coordination programs reduce hospital utilisation [17,18,19]. However, Australian research is limited and predominantly focuses on populations defined by their age group or chronic disease [6,9,20,21,22,23]. A noticeable gap exists in research exploring the impact of care navigation and coordination on a diverse population with varying medical conditions, age and psychosocial complexities.

This paper evaluates South Western Sydney Local Health District’s (SWSLHD) Planned Care for Better Health (PCBH) Program, a community-based care navigation and coordination program. We assess changes in hospital and Emergency Department (ED) utilisation at three months and six months before enrolling and after completing PCBH, and share key lessons learned for practice and policy. Through this, we hope to add to the existing body of knowledge and influence future policies for people with complex health and psychosocial needs in Australia.

### Ethical approval

Ethical approval was granted in 2023 by SWSLHD Human Research Ethics Committee (2023/ETH00220). This study involved analysis of regularly collected service-level data. Individual-level data was not reported, therefore waiver of consent was obtained.

## Description of the care practice: Planned Care for Better Health (PCBH)

**Figure 1:**
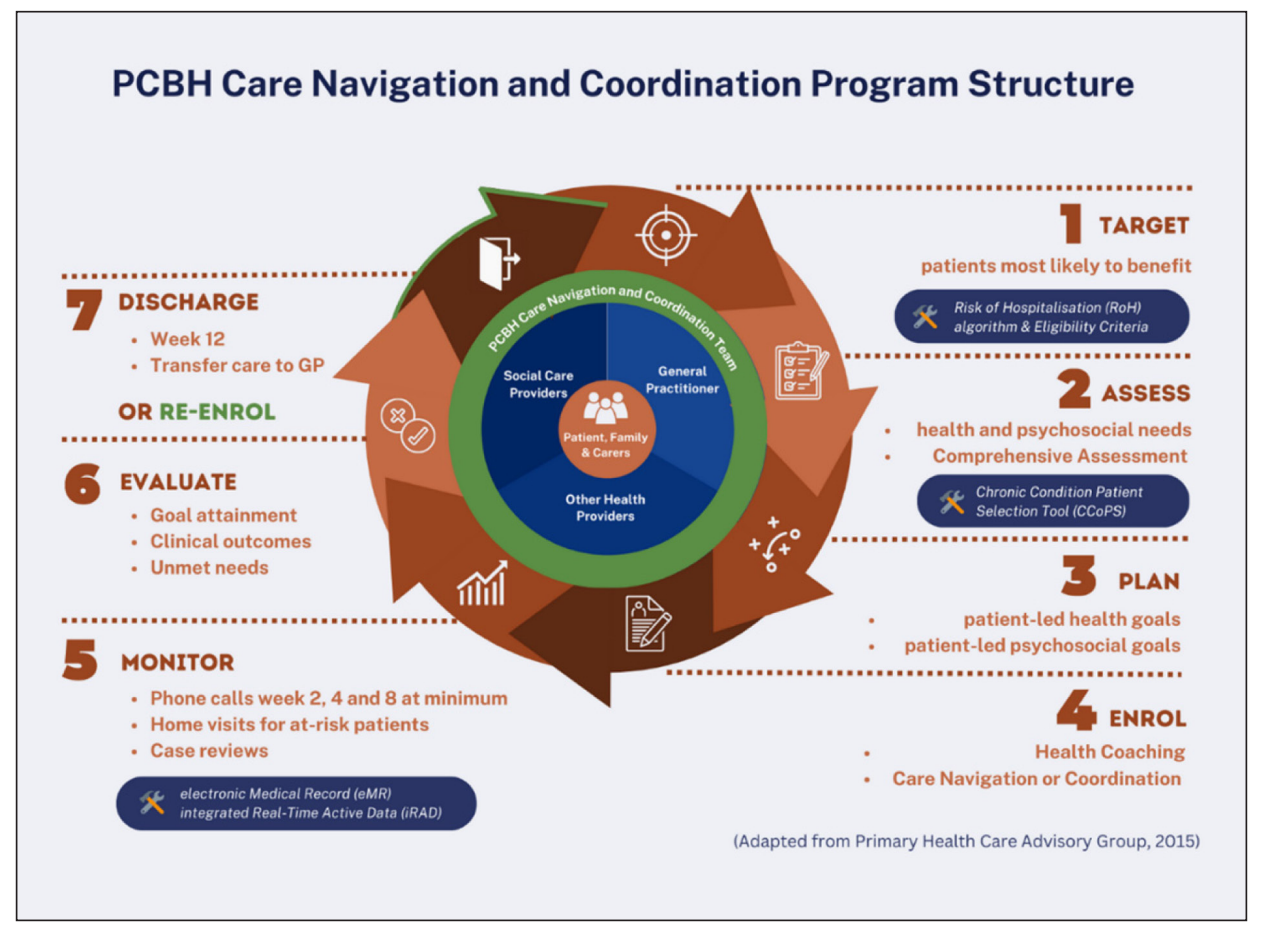
Planned Care for Better Health care navigation and coordination program structure.

PCBH is a 12-week community-based care integration program delivering person-centred care, promoting wellness, empowerment, and enablement by enabling integration of care through health coaching and care navigation or care coordination services (Figure 1). PCBH identifies clients at risk of future hospitalisation and aims to strengthen the care provided to them, with the goal of enhancing their care experience and reducing preventable hospital use. The program is delivered by care coordinators, comprised of Registered Nurses and Clinical Nurse Specialists with a variety of backgrounds and expertise, such as chronic care, rehabilitation, community health, geriatrics, mental health, drug and alcohol health, as well as from the acute and critical care settings. The nurses are supported by a project team who identifies, designs, and implements service-level quality improvements. All investigators of this study were involved in implementing PCBH in their respective roles as the Director, in care delivery or in project management.

### Implementation

#### Program Development

PCBH is the New South Wales (NSW) Ministry of Health flagship integrated care program supporting the Premier’s priority to improve outpatient and community care and reducing PPH visits by 5% by 2023. It is one of the state-wide key initiatives developed to foster communication and connectivity between primary, hospital and community health care providers and provide better access to community-based services closer to home. Whilst the Ministry of Health provided principles of implementation in the Planned Care for Better Health Transformation Plan, each Local Health District developed its own implementation and operational strategies (including workforce requirements and associated funding allocations) to suit its population needs. In SWSLHD, PCBH builds on its predecessor, the Integrated Care for People with Chronic Conditions (ICPCC) program by including a broader range of chronic conditions, as well as considering wider health determinants, psychosocial risk factors focusing on delivering outcomes that matter most to clients.

The development of PCBH is particularly impactful in the context of SWSLHD in which 49% of the residents reported having at least one chronic condition [24]. The number of ED presentation and PPH remain one of the highest in the state, accounting for 310,649 presentations and 22,079 hospitalisations respectively in 2020/2021 financial year [25,26]. Further, SWSLHD is one of the most culturally diverse regions in Australia, with 54.6% of residents speaking a language other than English at home. SWSLHD also welcomes the largest number of refugees and humanitarian entrants in NSW, with approximately 2,300 new arrivals settling in the region each year. It is home to a substantial population with profound or severe disabilities, with 1 in 15 individuals falling into this category, and 1 in 8 residents serving as caregivers for someone with a disability. Furthermore, SWSLHD reports higher levels of psychological distress compared to the overall NSW population [24]. The vulnerability of SWSLHD’s population also lies in the socioeconomic background, with a large portion of the population residing in Local Government Areas (LGAs) with higher-than-average levels of disadvantage compared to other LGAs in NSW [27].

#### Patient identification and enrolment

**Figure 2:**
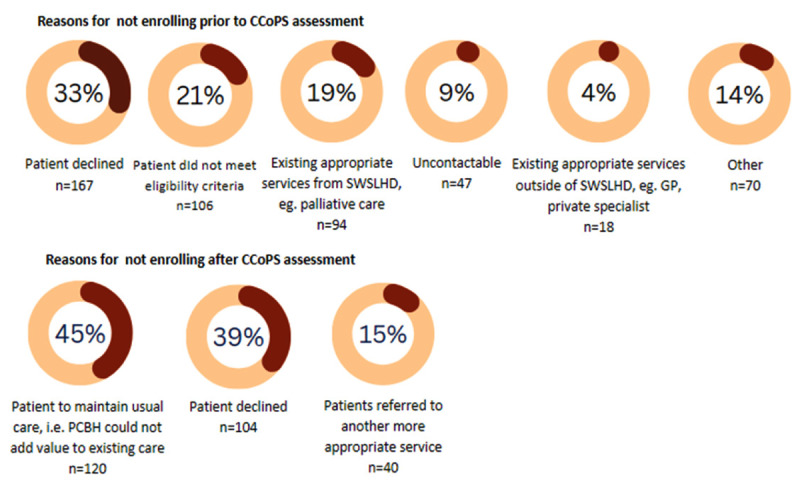
Reasons for not enrolling into PCBH program.

PCBH was initiated in July 2021 and is ongoing at time of writing. During PCBH’s early stages, the NSW Health Risk of Hospitalisation (RoH) algorithm was used to identify people at risk of hospitalisation in the next 12 months who would likely benefit from integrated care interventions [28]. The RoH algorithm is based on demographic and socioeconomic factors, as well as past public health service utilisation and medical history. In the Integrated Care module of the Patient Flow Portal (PFP) state-wide hospital administrative system, the RoH algorithm is applied to all patients who present to any public hospital in New South Wales. Following discharge Registered Nurse Care Coordinators filter this list and conduct desktop triage against the eligibility and exclusion criteria (Table 1). The care coordinator then calls those people deemed potentially eligible via phone to undertake a comprehensive assessment utilising the NSW Chronic Conditions Patient Selection (CCoPS) tool (Table 2). After confirming their eligibility and identifying their unmet health and psychosocial needs, enrolment to PCBH is offered. Reasons for not enrolling into the program are outlined in Figure 2.

**Table 1:** Eligibility criteria for PCBH enrolment and exclusion criteria.


Eligibility: adults age 16 years old or over residing in SWSLHD recent discharge from any NSW public hospital facility at least one chronic condition unmet health and/or psychosocial needs requiring navigation or coordination of care RoH score of 0.2 to 0.5 (excluding direct referral pathway) These criteria reflect the emphasis of PCBH in early identification of adults at risk of hospitalisation [28]. Exclusion criteria: Aboriginal and Torres Strait Islander background current inpatient Residential Aged Care Facility resident group home resident (criteria removed in 2023) correctional facility inmate DVA Gold Card holder who utilise its care coordination services The rationale of the exclusion criteria is that there are current care navigation and coordination services available for these population, for example the SWSLHD Aboriginal Chronic Care Program, and inpatient services from hospital, residential aged care, group home or correctional facility.


**Table 2:** Items included in patient assessments in week 1 and week 12 of intervention.


Comprehensive health and psychological assessments are completed using the NSW CCoPS tool, which assess the patient’s risk profile in the following domains. 1. Diagnosis or clinical symptoms of chronic conditions 2. Service access profile a. Hospital use in the past 12 months b. GP checks at least twice in the past 12 months c. Reduced ability to self-care impacting on disease management 3. Risk factors: a. Smoking b. Overweight/underweight c. Hypercholesterolaemia d. Hypertension e. Physical inactivity f. Polypharmacy 4. Potentially extenuating factors: a. Use of community-based services previously b. Carer stress issue c. No carer d. Cognitive impairment e. Recent change to drug regimen f. Chronic pain g. Compromised skin integrity h. Exposure to triggers for asthma 5. Patient self-reported health status 6. Psycho-social and demographic factors a. Mental health issues b. Disability c. Difficulty accessing transport to services d. Financial issues e. Aboriginal or Torres Strait Islander status f. Culturally and linguistically diverse g. Illiteracy and/or limited English h. Unstable living environment i. Social isolation j. Drug and/or alcohol problems k. Impact rating on self-management ability 7. Patient’s readiness to change.


As the program matured, direct referral pathways with other care providers were developed, allowing PCBH to accept patient referrals from SWSLHD community nursing teams, SWSLHD and other Local Health District hospitals, Justice Health and Forensic Mental Health Network, General Practitioners, as well as self-referral for people residing within the boundaries of SWSLHD, enhancing efficiency and integration of patient care [29]. The eligibility criteria for these people remains the same, except that the RoH score is not considered. The first patient enrolled through a direct referral pathway occurred in September 2022. In 2023, group home residents were no longer excluded due to identified needs.

#### Intervention

Through PCBH, clients, families and carers receive holistic health coaching, and care navigation or coordination services, focusing not only on medical aspects but also psychological, social, and behavioural factors (Table 3). The care coordinators include client, family and carers in planning their care, listening to their needs, supporting them to achieve their goals and empower them to make decisions on their own health. In partnership with the patient, their families and carers, the care coordinators identify gaps in care, develop goals, provide education, connect the patient with relevant health and social care providers, and improve client’s self-management skills. By removing barriers to healthcare, we empower clients to manage their health conditions in the community. Client contact and intervention are delivered mostly via phone calls. Home visits may be provided for care coordination clients as it has been shown to be effective for vulnerable and complex clients [1]. Care coordinators also work closely with the client’s General Practitioner and care providers, such as medical specialists, allied health practitioners, behavioural therapists, disability care coordinators, and social support services. Collaboration is conducted virtually or in-person, via case conferences, case reviews, emails, letters, and phone calls.

**Table 3:**
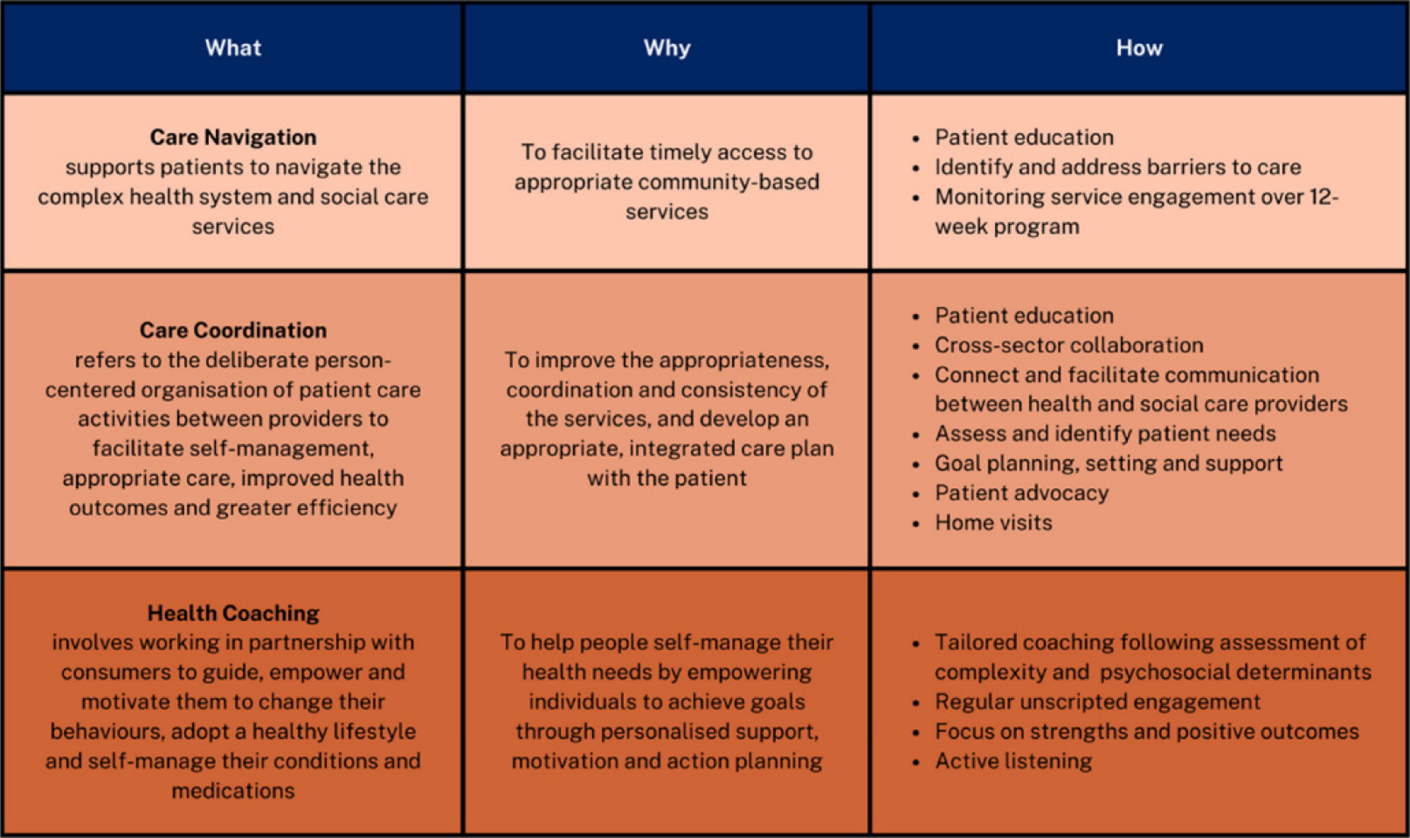
PCBH interventions.

There was a notable effort in nurturing relationships with clients, families and carers. Some clients displayed reluctance to engage due to prior negative encounters with the healthcare system. The virtual nature of PCBH introduced a unique challenge, as unexpected initial phone contact caused surprise and reluctance for some clients to engage with the program. Through a series of positive engagements over the first weeks of enrolment that acknowledge clients’ experiences, apprehending their concerns, and consistently showcasing a commitment to their overall wellbeing, the clinicians have focused on the development of trusting therapeutic relationships, which is associated with success with clients [30].

Table 3 distinguishes between the three different intervention types offered in PCBH. Care Navigation is suitable for clients who are able generally able to self-manage but require additional support to navigate the complex health system and social care services. Care Coordination is suitable for more complex clients who require additional support to self-manage and coordinate their care. Health Coaching is frequently offered in addition to Care Navigation and Care Coordination and involves working in partnership with clients to guide, empower and motivate them to change their behaviours and self-manage their conditions.

#### Monitoring and discharge

Clients’ health status was monitored through a combination of methods, such as follow-up phone conversations with clients and associated care providers. Desktop file reviews were conducted utilising the electronic Medical Record (eMR) and PFP to observe hospitalisation events. Furthermore, Integrated Real-Time Active Data was used to access real-time insights into various occurrences, such as visits to General Practitioners, pathology reports, care management plans, and medication regimes. Daily risk huddles and monthly case reviews facilitate discussions between the clinical team and senior clinicians, aiding in the formulation of care plans for clients at risk of deterioration.

Follow up with the client occurs at week 2, 4, and 8 at a minimum, with more arranged as needed. At week 12, clients were re-assessed using the CCoPS tool. If additional care management is required, their care plan is reviewed in consultation with the patient and clinical team, and enrolment can be continued until their needs are met (less than 5% of all enrolments). If their health and psychosocial needs have been met, clients are discharged and return to usual care managed by their General Practitioner. PCBH phone contact details are provided to clients so they can re-engage if needed.

#### Quality improvements

Ongoing quality improvement activities were undertaken to address identified gaps and improve client and clinician experiences. Some examples were regular collation of information pertaining to local services and referral options; development of a variety of work instructions and templates to ensure consistency in care delivery; implementation of text messaging before initial phone call to improve engagement and ensuring clients are aware of their rights and responsibilities as a patient.

#### Illustrative case stories

Below are two accounts of clients who have completed PCBH. These narratives demonstrate the comprehensive nature of their health and psychosocial needs, as well as the collaborative effort from various agencies needed to support the clients, their families, and caregivers. *Names have been altered to maintain confidentiality.

##### Case story 1 – care coordination

Joe*, was frequently visiting the ED due to fears about illnesses he had read about on the internet. His undiagnosed and unmanaged intellectual disability led to significant behavioural issues, affecting his social interactions. Joe lives with his elderly mother and younger sister, and he has been refusing to see any health practitioners. Joe’s challenging behaviour often created a tense home environment. Joe’s mother does not speak English and she is unable to operate a computer or the internet. Through care coordination, the PCBH program initiated a collaborative intervention with his General Practitioner, social worker, specialist intellectual disability health team, National Disability Insurance Scheme (NDIS) local area coordinator, and mental health team. Through home visits, the necessary assessments for Joe’s NDIS application were completed. Emotional and practical support was extended to his family, as well as education on disability and mental health. As a result, Joe obtained a comprehensive NDIS plan, inclusive of support for household activities, community involvement, respite and ongoing reviews by a Behavioural Therapist, an Occupational Therapist and a Speech Pathologist. After his NDIS plan was implemented and Joe was discharged from PCBH, Joe had no further ED visits at three months and six months, indicating the effectiveness of the collaborative and comprehensive approach of the PCBH program.

##### Case story 2 – care navigation

Sam*, has had hospital readmissions with fevers and deconditioning. He had multiple chronic conditions and he was dependent on his wife for all activities of daily living. Sam had issues with incontinence and a pressure ulcer, as well as being at high risk of dysphagia, aspiration and falls. As the carer, Sam’s wife also had high levels of stress due to Sam’s increased acuity, and reduced domestic assistance received in the context of the COVID outbreak at the time. Through PCBH, Sam and his wife were provided with education about current health concerns and their risks. A management plan was also developed. The care coordinator guided Sam’s wife to have the pressure ulcer reviewed by the General Practitioner, and to utilise Sam’s Home Care Package to access domestic assistance. The care coordinator encouraged ongoing follow up with Speech Pathology and initiated referral to Physiotherapy for a Falls Intervention Program. Sam’s wife was also supported through by Carer Gateway program. Sam and his wife highlighted that they felt listened to, were grateful for the program, and are now aware of how to get in contact with support services to follow up should further concerns arise.

## Evaluation

**Figure 3:**
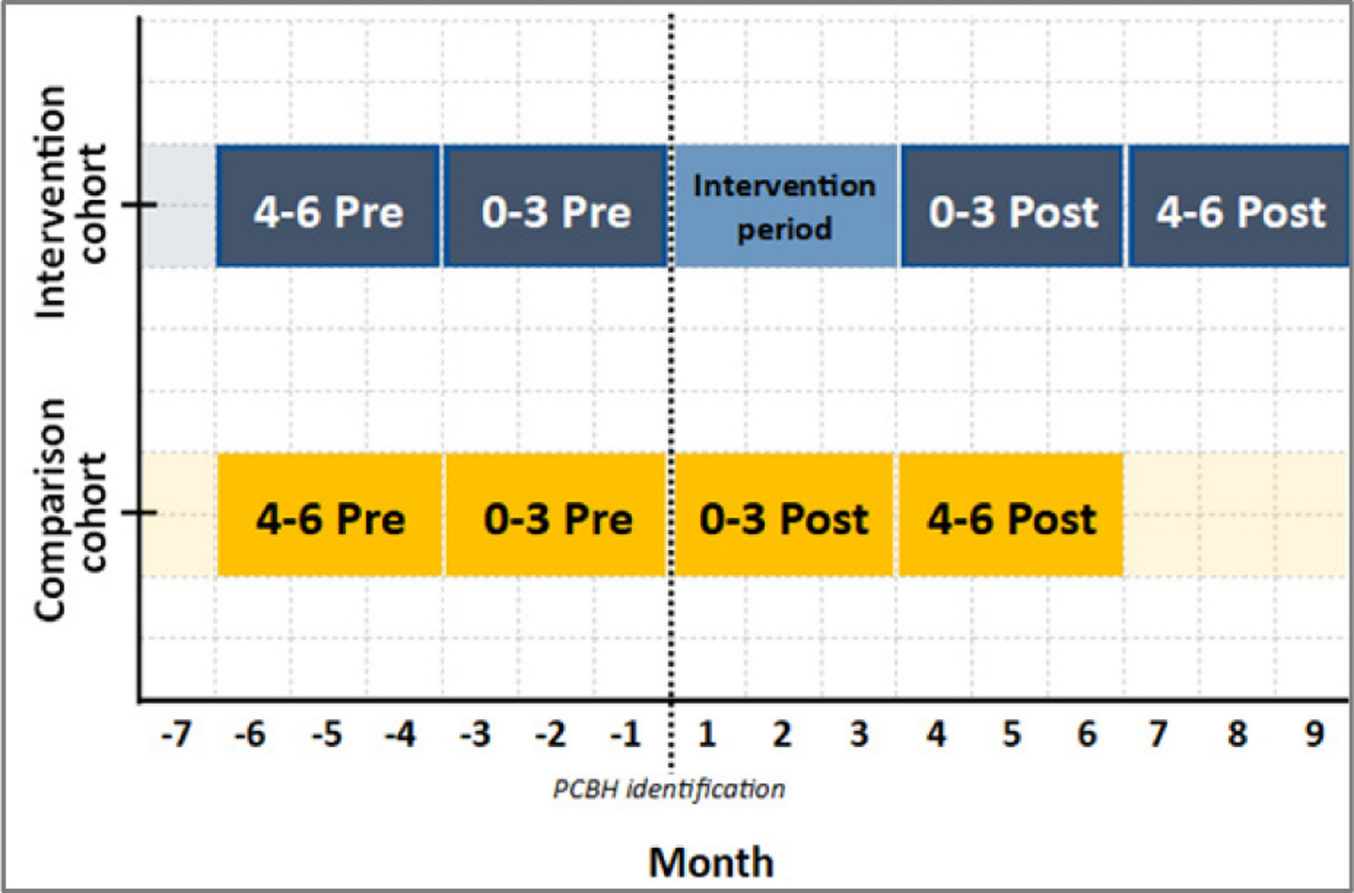
Demonstration of study periods.

Proactive outcome monitoring began in September 2021, where a project officer began collating routinely collected demographic and outcome measures data from clients’ eMR and from SWSLHD’s routine administrative system of PFP, for the purposes of reporting, progress tracking and program evaluation. The change of hospital use, at 0–3 months and 0–6 months before enrolment into PCBH versus after completion of the PCBH program, of the intervention group was compared to the change in the comparison group (Figure 3). Six months’ follow up data were used to inform sustainability of outcomes.

### Methodology and Study Population

With retrospective observational cohort evaluation study design, we have analysed data associated with PCBH clients between 1st September 2021 to 31st July 2022. This study period was selected to allow adequate time for six months of post enrolment outcome monitoring. Utilising non-probability systematic sampling, clients were sampled at regular intervals until the maximum sample size, as workload allowed, was achieved.

**Figure 4:**
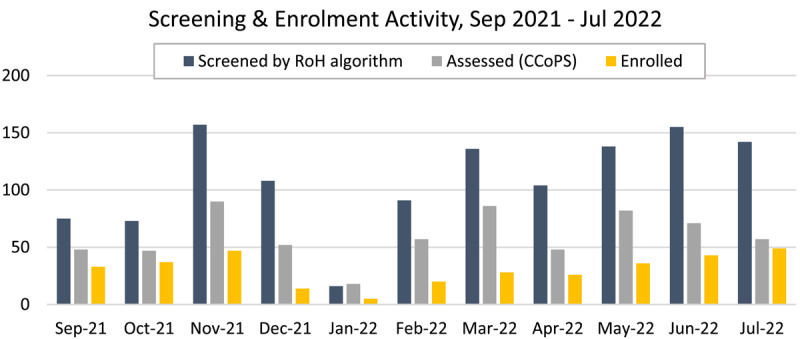
Screening and enrolment activity.

Between September 2021 and July 2022, there were 1,195 clients screened, resulting in 319 clients consenting to program enrolment (Figure 4). From the 319 enrolled clients, 60 clients who completed the 12-week PCBH program and were subsequently discharged between the above dates are included in the intervention cohort. Meanwhile, 60 people who were eligible to enrol but declined enrolment to the program between the above dates are included in the comparison cohort. Any clients who died or transitioned to a residential aged care facility were excluded from analysis.

### Outcome measurements and data analysis

Outcome measurements for hospital utilisation include: (i) number of unplanned hospitalisations, (ii) total bed days, (iii) average length of stay, and (iv) number of ED presentations. Unplanned hospitalisation is defined as a hospital admission that is unexpected and not scheduled in advance. Whilst all PPH are unplanned, not all unplanned hospitalisations are potentially preventable. The unplanned hospitalisation measure was selected as it provides a clear and objective measure in comparison to PPH whose criteria has changed over time and varies between regions [31]. All measurements were analysed using difference-in-difference models. Datasets accessed include routinely collected data available from SWSLHD, such as eMR and PFP. Each patient was assigned an individual subject study number. Data collection and statistical analysis were conducted on the de-identified database.

For ED presentations, only the date of arrival was analysed. Whilst for unplanned hospitalisations, dates of admission and discharge, principal diagnosis and length of stay were analysed. Hospital admissions for procedures previously planned, such as a gastroscopy or a planned infusion, are excluded from analysis.

For the purpose of this study Hospital utilisation outcomes measures for six months pre-enrolment were compared to Hospital utilisation outcomes measures six months post-enrolment. The same time periods were used for the control cohort to allow comparison of outcomes between groups.

### Participants characteristics

**Figure 5:**
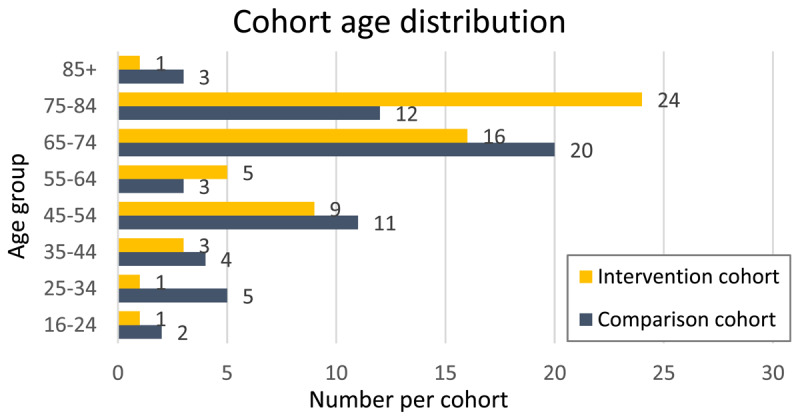
Cohort age distribution.

Study participants’ demographics, health and psychosocial characteristics are summarised in Table 4. The most frequent diagnoses in both cohorts were cardiac conditions and chronic pain. The three primary vulnerabilities identified were mental health or drug and alcohol issues, cultural and linguistic diversity (CALD), and disability. The dominant age groups for both cohorts were 65–74 and 74–84 years (Figure 5). Within the intervention cohort, 87% (52 clients) received care navigation, whilst 13% (8 clients) received care coordination.

**Table 4:** Participants demographic, health and psychosocial characteristics.


Characteristics	Intervention cohort (n = 60)	Comparison cohort (n = 60)

Age – Mean ± SD	69 ± 15	61 ± 18

Sex (%)		

Male	55.0%	51.7%

Female	45.0%	48.3%

Country of origin (%)		

Australia	46.7%	50.0%

Overseas	53.3%	50.0%

Primary language spoken (%)		

English	65.0%	85.0%

Non-English	35.0%	15.0%

Chronic Disease (n)		

Chronic Cardiac Condition	38	38

Chronic Pain	36	39

Diabetes, Renal Failure and/or Liver Disease	33	20

Chronic respiratory issues	27	20

Compromised skin integrity, e.g. wounds	12	12

Vulnerability (n)		

Mental health and/or drug & alcohol issues	35	33

CALD background and/or limited English	35	32

Mental and/or physical disability	30	35

Financial issues	8	2

Social isolation	4	4

Risk factors and lifestyle concerns (n)		

Lives in socio-economic disadvantaged area	49	50

Overweight, obesity or underweight	31	25

Dementia, falls, or incontinence	30	21

Hypercholesterolaemia	27	31

Reduced ability to self-care	26	20

Recent change to drug regimen	22	26

Physical inactivity	20	12

Smoking	10	9

Carer stress and/or no carer availability	13	23


### Hospital Utilisation Outcomes

The changes in hospital utilisation for both intervention and comparison cohorts are summarised in Table 5.

**Table 5:** Hospital utilisation before and after PCBH program.


	UNPLANNED HOSPITALISATIONS		TOTAL BED DAYS		AVERAGE LENGTH OF STAY (DAYS)		ED PRESENTATIONS	

	INTERVENTION	COMPARISON	INTERVENTION	COMPARISON	INTERVENTION	COMPARISON	INTERVENTION	COMPARISON

**0–3 months pre-PCBH program enrolment**	77	61	444	253	5.218	4.48	79	67

**4–6 months pre-PCBH program enrolment**	33	20	104	113	3.486	5.889	56	49

**Total pre (0–6 months)**	110	81	548	366	8.704	10.369	135	116

**0–3 months post-PCBH program completion**	52	40	126	151	3.754	4.009	30	30

**4–6 months post-PCBH program completion**	33	35	174	159	5.492	5.063	42	18

**Total post (0–6 months)**	85	75	300	310	9.246	9.072	72	48

**Reduction pre-post (n) at 0–3 months**	25	21	318	102	1.464	0.471	49	37

**Reduction pre-post (%) at 0–3 months**	32.5%	34.4%	71.6%	40.3%	28.1%	10.5%	62.0%	55.2%

**Reduction pre/post (n) at 0–6 months**	25	6	248	56	–0.542*	1.297	63	68

**Reduction pre-post (%) at 0–6 months**	22.7%	7.4%	45.3%	15.3%	–6.2% *	12.5%	46.7%	58.6%


note that *indicates an increase in hospital use rather than a decrease.

Unplanned hospitalisations in the intervention cohort reduced by 32.5% at three months post intervention, and 22.7% at six months post intervention. This is a more sustained outcome than the comparison cohort, where unplanned hospitalisation reduced by 34.4% at three months and 7.4% at six months.

Total bed days for unplanned hospitalisations in the intervention cohort reduced by 71.6% at three months post intervention, and 45.3% at six months post intervention. This is a greater reduction than the comparison cohort, where total bed days for unplanned hospitalisation reduced by 40.3% at three months, and 15.3% at six months.

The average number of bed days per unplanned hospitalisation in the intervention cohort reduced by 28.1% at three months post intervention, yet increased by 6.2% at six months post intervention. In contrast, the comparison cohort maintained a reduction at both time points, with 10.5% at three months and 12.5% at six months.

ED presentations in the intervention cohort reduced by 62.0% at three months post intervention, and 46.7% at six months post intervention. ED presentations in the comparison cohort reduced by 55.2% at three months, and 58.6% at six months.

## Discussion

This retrospective analysis has evaluated hospital utilisation outcomes of clients with complex health and psychosocial needs who completed the 12-week PCBH care navigation and coordination program in SWSLHD, Australia.

Both cohorts showed a reduction in unplanned hospitalisations at three months. Yet, at six months, the intervention cohort’s reduction was notably better than the comparison group. This analysis is consistent with current literature. Care navigation and coordination contributed to reducing PPH through facilitation of timely and effective continuity of care [22], improving symptom monitoring [32] and increasing interdisciplinary communication [19,33]. Existing literature also highlights the important role that care coordinators have in partnership-building between care providers which, in a background health system culture of non-integration, requires work to develop and maintain [1,23]. This proactively fosters collaborations with multiple clinical and psychosocial care providers, as well as building their capacity to provide appropriate care to the clients [6].

The importance of addressing psychosocial needs is consistent with the existing evidence, highlighting socioeconomic needs as key drivers to medical outcome and costs [5,6,11], and that addressing them is central to improving both [1,34]. This notion is central to the holistic approach adopted by PCBH which looks beyond the disease or the intervention to focus on individual needs [14]. Acknowledging psychosocial needs through mutual respect between clients and care coordinators has been linked with improved care satisfaction and better outcomes [34]. A relatively high proportion of PCBH clients enrolled lived in areas classified as socioeconomically disadvantaged, which is associated with avoidance or delay in seeking or receiving medical care and increased PPH rate [11,35]. Clients with complex health needs, residing in areas where poor health and socioeconomic insecurity are deemed acceptable social norms, require such navigation and coordination support [36].

The PCBH program is associated with noteworthy reductions in the total bed days at three and six months when compared to the comparison group. The intervention cohort released 248 bed days at six months post-completion of PCBH, compared to the comparison cohort’s 56 released bed days. Bed days, as an element of healthcare cost analysis, represents economic potential when released for use by other clients who require hospitalisation, or different healthcare applications [37]. Emphasising this concept of opportunity cost, PCBH fosters sustainable efficiencies within healthcare systems through optimisation of resource allocation [38]. The intervention cohort demonstrated an increase in the average length of stay per unplanned hospitalisation. The research team speculated that this might be due to higher patient acuity upon presentation, necessitating longer hospital stays. This may indicate that clients are accessing hospital services appropriately and when needed, however, further analysis is needed to substantiate this observation.

The intervention cohort had considerable reduction in ED presentation at the three months mark yet this was not sustained at six months. In contrast, the comparison cohort demonstrated steady ED reduction rates throughout the study period. Previous studies have shown mixed impact of care navigation and coordination programs on ED presentations. Whilst some studies highlighted positive impact [13,21,22], it has been suggested that through care navigation and coordination, the clients may find an unmet health need or deterioration, hence increased ED presentations [23,39,40]. ED presentations may also be influenced by factors beyond care navigation and coordination programs such as limited access to primary care and availability of appointments, which need addressing [13,41]. Patient factors such as age, socioeconomic status, education, perceptions of primary healthcare [41], social isolation and a preference for comprehensive ED services [13] further drive ED attendance. Yet, the trend observed toward decreased ED presentation at three months following PCBH program completion is encouraging. This may likely be due to the intensive support provided to clients through the program [13], hence at three months clients experienced significant improvements in their health outcomes. Further investigation is needed to determine the specific aspects of care coordination are most successful and needed to ensure sustainability of outcomes after 6 months.

With complex needs, shifting health and psychosocial situations often impact on clients’ prioritisation and self-management skills [1,42]. Therefore, it is important to highlight that the goal of care navigation and coordination programs extends beyond the immediate health improvement, and instead these programs aim to empower clients with skills and knowledge they need for long-term self-management [14] and develop their confidence to engage services when faced with challenges. This way, the program’s impact can reverberate far beyond its completion, promoting healthier and more empowered communities.

### Strengths and Limitations

To our knowledge this is the first study in Australia to evaluate the impact of a care navigation and coordination program on hospital utilisation in a diverse study population with unmet psychosocial needs. Unlike other studies in which participants had age-specific or disease-specific criteria, the PCBH cohort are diverse in their chronic conditions, age, cultural, linguistic, and socioeconomic backgrounds.

Limitations of this study include its retrospective design, where retrospective client experience feedback and creation of a control group was not possible. The study also utilised an unmatched comparison group. However, we believe analysing the two groups provided valuable insight into the impact of PCBH program. Larger sample size in a randomised controlled trial would have allowed confounding factors, including health and psychosocial provision, to be evenly distributed between the groups.

## Lessons Learned – Implications for Policy and Practice

### Leadership was key in achieving shared understanding of integrated care

Integrated care, as a concept, often elicits a variety of interpretations. Whilst engaging different stakeholders, clients, clinicians, care providers, executives, and the broader community, we noted that having a clear vision with shared value was crucial. A consistent visionary leader is vital to navigate periods of change, ensuring continued engagement due to the particularly complex nature of integrated care where multiple organisations and professionals are involved in the care of a patient [43]. The leadership changes at the very beginning of PCBH’s implementation, as well as the absence of an operational management role for the service, contributed to the uncertainty in strategic and operational direction. These factors potentially hindered the programs progress and its perceived legitimacy from internal and external stakeholder viewpoints. This situation underscores the significance of consistent and visionary leadership for maintaining program stability and enhancing stakeholder confidence [44,45]. We learned that having this consensus not only encouraged collaborative actions, streamlined decision-making, and fostered efficient communication, but also ensured alignment with integrated care culture and achievement of desired outcomes [46]. Moreover, a shared understanding promoted transparency and trust, inviting collective engagement critical for transforming the healthcare landscape into a system that’s interconnected, responsive, and ultimately patient-centred [43].

### Early engagement of service level staff to build direct referral pathways

More thorough engagement with health facilities and the development of direct referral pathways within the planning phase would have improved engagement by health facilities and should be considered in any future iterations of the model of care. While PCBH benefited from an executive steering committee comprising numerous executive leaders and senior directors, the representation of facility or patient needs could be further enhanced. Participative co-production with key hospital personnel, such as nurse unit managers, senior allied health clinicians and chronic disease educators, could provide better insights into the support these providers require in the delivery of everyday care. This may enhance their participation and program’s transferability and applicability [47]. The early establishment of simple and coordinated pathways that allow clinicians to expedite the cases of clients who urgently require services has been recommended to enhance the quality of patient outcomes and strengthen the primary care sector [5,15], yet this practice could be improved in Australia [35,48]. Our ability to promote PCBH referral pathways during the early stages of implementation was limited by the associated uncertainty arising from temporary program establishment pending demonstrable outcomes. This temporary status introduced challenges in forming enduring partnerships with health facilities and subsequent establishment of direct referral pathways, given the necessity to manage expectations and deliver within a limited timeframe.

### Multidisciplinary care team may improve program capability

Enhancing the workforce by Including allied health roles, such as social workers and occupational therapists, would enhance the multidisciplinary nature of the team and enhance clinical expertise associated with the delivery of care navigation and coordination to better support the complex needs of enrolled clients. During the planning period, while the goal was to include these roles in the team, due to diverse priorities and viewpoints, the present composition includes only nursing staff. We saw a noticeable limitation of availability of allied health services in the District. Incorporating allied health professionals directly within PCBH could potentially overcome access constraints, offering clients a more seamless, efficient, and comprehensive service [49].

### Early creation of robust evaluation plan to improve the quality of data

An early and dedicated focus on the creation and execution of a robust evaluation and research plan would be invaluable to understand impact, demonstrate outcomes and justify ongoing funding [44]. The absence of rigorously collected statistical data and matched control group limited the program’s ability to accurately showcase the impact that the intervention had on hospital utilisation outcome measures. This was largely due to a lack of resources and capacity to manage such an initiative at the time, as well as the team’s reliance on data capture and monitoring systems outside of their influence. However, greater control over these processes from the outset could improve the quality and accuracy of the data to extract meaningful insights, fostering data-driven decision-making to improve healthcare outcomes.

## Conclusion

Integrated care is essential in Australia in response to the rise of chronic diseases and the complexities these clients face, including psychosocial challenges and health system navigation difficulties. The current healthcare system in Australia is siloed and complex, leading to uncoordinated care, service delays, and increased vulnerability, with chronic conditions resulting in high potentially preventable hospitalisations costs [8].

The PCBH Care Navigation and Coordination Program helps to bridge gaps in the healthcare system for those with the greatest vulnerabilities. The PCBH program aims to identify clients with complex health and psychosocial needs at risk of hospitalisation early, delivering targeted intervention and strengthening the care provided to them, with the goal being to improve their experience and reduce preventable hospital utilisation. PCBH correlates with a notable decrease in unplanned hospital stays and overall bed days, yet the decrease in ED visits is comparable between the intervention and comparison groups.

Although Australia has championed various integrated care policies, there remains room for clinical practices to better align with integrated care principles, practices and policies particularly in relation to partnering with clients and better considering their psychosocial needs. Future care navigation and coordination efforts must employ clear, shared vision of integrated care, robust leadership, and participative co-creation with service-level stakeholders. Additionally, securing ongoing service funding, establishing enduring partnerships with health facilities and implementation of direct referral pathways with these facilities, establishing a multidisciplinary team of care coordinators and early establishment of an evaluation framework could prove to be crucial.
